# Comprehensive Investigation of *Moringa oleifera* from Different Regions by Simultaneous Determination of 11 Polyphenols Using UPLC-ESI-MS/MS

**DOI:** 10.3390/molecules25030676

**Published:** 2020-02-05

**Authors:** Yanqin Zhu, Qinhong Yin, Yaling Yang

**Affiliations:** 1 Faculty of Life Science and Technology, Kunming University of Science and Technology, Kunming 650500, China; 2 Research Center for Analysis and Measurement, Kunming University of Science and Technology, Kunming 650093, China; 3 Faculty of Narcotics Control, Yunnan Police College, Kunming 650223, China

**Keywords:** polyphenols, different regions, UPLC-ESI-MS/MS, *Moringa oleifera*

## Abstract

In this study, we develop and validate a simultaneous quantification of polyphenols method based on an ultra-performance liquid chromatography-electrospray ionization-tandem mass spectrometry (UPLC-ESI-MS/MS) to adequately understand how different habitats influence the quality and profile of *Moringa oleifera* polyphenol. Furthermore, principal component analysis (PCA) and hierarchical cluster analysis (HCA) were used to compare and discriminate 25 samples collected from different areas. A significant correlation was found between the polyphenol profile and the collection area. Significant differences in the polyphenol content of *Moringa oleifera* from different regions indicate that the genetic diversity of *Moringa oleifera* was relatively rich, possibly due to differences in cultivation conditions, climate, or soil environment resulting in the accumulation of different polyphenols. These observations provide a theoretical basis for subsequent *Moringa oleifera* germplasm selection and development research. Furthermore, the quantitative analysis methodology used to characterize the polyphenols may be used toward developing quality assessment and future pharmacodynamic investigations of *Moringa oleifera*.

## 1. Introduction

*Moringa oleifera* belongs to the family of Moringaceae, that is widely distributed throughout the world, especially in Asian and African countries [1,2,3]. Within this family, *Moringa oleifera* is the most widely cultivated and studied species [4]. Due to its edible leaves, roots, fruits, flowers, and nutritious pods, it can be utilized as a food, nutraceutical, and medicine. Therefore, *Moringa oleifera* is called the “miracle tree” [5,6].

Studies have demonstrated that *Moringa oleifera* has a great number of bioactive compounds [7,8] that have beneficial effects in humans [9]. However, the most used parts of the plant are the leaves, which are mainly rich in potassium, calcium, phosphorous, iron, protein, vitamins A, C, and E, β-carotene, carotenoids, polyphenols [10,11,12,13,14,15,16,17,18], oxidase, catalase, alkaloids, glucosinolates, isothiocyanates, tannins, and saponins [19,20,21,22]. A large number of bioactive substances may account for its medicinal properties, which include hypotensive, anticancer, antioxidant, antibiotic, antiulcer, anti-inflammatory, skin infection, anemia, cuts, scrapes, rashes, signs of aging, malaria, typhoid fever, diarrheal, dysentery, colitis hepatoprotective, hypocholesterolemic, and hypoglycemic activities [23,24,25,26,27,28,29,30]. Consequently, *Moringa oleifera* leaves are used as a kind of functional food for human nutrient fortification in both developing countries and developed countries.

The polyphenol content of *Moringa oleifera* differs according to plant part. Due to drought stress and cumulative effects, the dried leaves of *Moringa oleifera* are rich in polyphenols, of which flavonoids and phenolic acids are the principal compounds. The observed flavonoids are mainly quercetin and kaempferol [6], while the phenolic acids are mainly gallic, chlorogenic, ellagic, and ferulic acids. Flavonoids effectively remove oxygen free radicals in the body and ameliorate cardiovascular and cerebrovascular diseases [31]. Phenolic acids have antioxidant and antitumor functions [32]. At present, *Moringa oleifera* is widely cultivated in Yunnan province in China [3], Due to various complicated environmental factors, the nature of polyphenols found in *Moringa oleifera* varies greatly, though information regarding the polyphenol profiles of plants in different areas of Yunnan province remains limited. Therefore, to ensure maximum biological efficacy, the variance in contents of major polyphenol compounds in *Moringa oleifera* among different production areas should be further investigated.

By surveying the relevant literature, it was determined that different analytical methods have been used for the study of polyphenols in *Moringa oleifera*. These include high-performance liquid chromatography with ultraviolet detection coupled with mass spectrometry (HPLC-UV-MS) [21], high-performance liquid chromatography with ultraviolet detection (HPLC-UV) [33,34], liquid chromatography-tandem mass spectrometry (LC-MS/MS) [35], and ultra-performance liquid chromatography-quadrupole time of flight mass spectrometry (UPLC-QTOF/MS) [36]. From the above analysis, the simultaneous determination of polyphenols in *Moringa oleifera* by ultra-performance liquid chromatography-electrospray ionization-tandem mass spectrometry (UPLC-ESI-MS/MS) has not yet been conducted.

UPLC-ESI-MS/MS is a rapid, sensitive and selective technique, that can save time, and use less solvent, while providing accurate determination of compounds. Therefore, the aim of this study was to establish an UPLC-ESI-MS/MS method for the simultaneous determination of 11 phenolic components in *Moringa oleifera*, in order to improve the quality control of *Moringa oleifera*. Moreover, the differences in contents of 11 polyphenolic compounds corresponding to plants of different regions was comprehensively investigated and evaluated with principal component constituent analysis (PCA) and hierarchical cluster analysis (HCA).

## 2. Results and Discussion

### 2.1. Optimization of UPLC and MS Conditions

**Figure 1:**
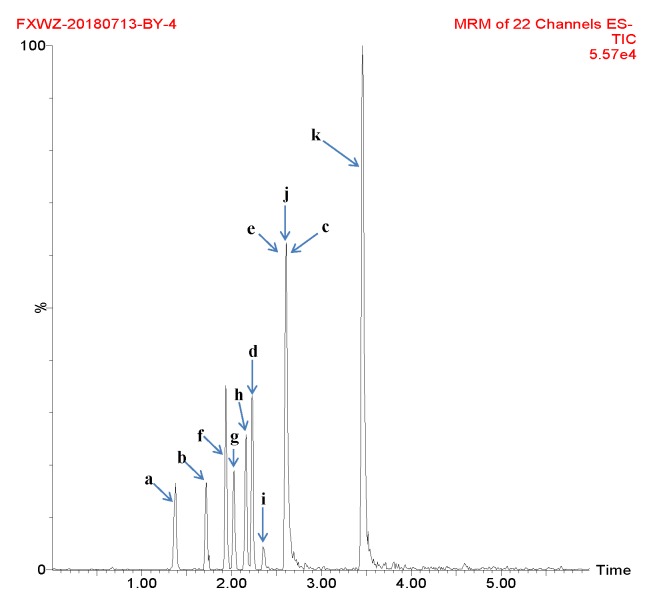
Total ion chromatogram (TIC) of compounds in a representative sample (a: gallic acid; b: chlorogenic acid; c: vanillin; d: ferulic acid; e: gallogen; f: rutin; g: isoquercetin; h: quercitrin; i: baicalin; j: quercetin; and k: kaempferide).

The mobile phase, elution mode, flow rate and chromatographic column for UPLC were selected to fully obtain the optimal signal strength of each compound. A mobile phase consisting of methanol/water or acetonitrile/water in various proportions did not result in any specific improvements in peak shape, through a mobile phase of acetonitrile and 0.1% formic acid aqueous solution produced peaks with better symmetry and with good ionization of analytes. Different columns were used for separation, including ACQUITY UPLC BEH C18 (50 × 2.1 mm, 1.7 µm), ACQUITY UPLC BEH C18 (100 × 2.1 mm, 1.7 µm) and Agilent Eclipse Plus (50 × 2.1 mm, 1.8 µm). It was shown that the best peak shape resulted from the ACQUITY UPLC BEH C18 (100 × 2.1 mm, 1.7 µm) column. The column temperature was set at 30 °C to gain good chromatographic peaks. Figure 1 shows the total ion chromatogram (TIC) of compounds in a representative sample.

Generally speaking, the charged analytes in solution could be detected more sensitively using ESI mode. Nevertheless, the atmospheric pressure chemical ionization (APCI) was usually used for the analysis of uncharged matter in solution. Polyphenols had many hydroxyl groups and tended to form charged substances. In order to obtain maximum sensitivity for the identification and detection of analytes, electrospray ionization with positive ionization mode (ESI^+^) and negative ionization mode (ESI^−^) were selected. After infusion of 100 ng/mL standard solution, the 11 polyphenols produced stable and strong signal precursors [M − H]^−^ at 150.99–609.10 *m*/*z*. After fragmentation by collision gas, precursors produced signals of the largest products at 106.99–300.42 *m*/*z*. The second largest products were simultaneously produced at 79.07–275.00 *m*/*z*. As a result, the signal corresponding to the largest product was viewed as the quantifier, and that of the second largest product was regarded as the qualifier. Table 1 lists the multiple reaction monitoring (MRM) conditions of 11 polyphenols using tandem quadruple detector-mass spectrometry (TQD-MS). Figure S1 lists precursors of 11 polyphenols in TQD-MS.

### 2.2. Optimization of Sample Extraction Procedure

Satisfactory extraction efficiency was achieved for sample extraction by comparing water, water/methanol (70/30), water/methanol (50/50), water/methanol (30/70) and methanol. According to total content of the 11 polyphenols, high total content was obtained with methanol. Therefore, methanol was selected as the solvent for extraction of *Moringa oleifera*. Meanwhile, different extraction methods were compared, such as soak extraction, ultrasonic extraction, reflux extraction and Soxhlet extraction, but as there were no significant differences in extraction rate, ultrasonic extraction was chosen for its simplicity and convenience. Moreover, different extraction times of 20, 40, 60, and 100 min were investigated for ultrasonic extraction of *Moringa oleifera* samples. No effective improvements in extraction rate were observed for extraction times over 40 min. The ultrasonic extraction temperature was set at 20 °C because high temperatures can accelerate the oxidation of polyphenols. Consequently, the optimal extraction conditions were determined to be ultrasonic extraction with methanol for 40 min at 20 °C (Figure S2).

### 2.3. Method Validation

The method was validated by determining matrix effects, linearity, limit of detection (LOD), limit of quantitation (LOQ), precision, accuracy and ruggedness according to the “International Conference on Harmonization (ICH)” guidelines.

According to a previous report [37], a fairly complete description of the matrix effects can be achieved by acquiring calibration plots with three sample sets. The first sample was a *Moringa oleifera* leaf sample, the second was a standard solution at 0.5 μg/mL, and the third was a sample spiked to a concentration of 0.5 μg/mL. The matrix effect can be evaluated using from the following equation, where “A” represents the area of the corresponding sample.

$$\mathrm{Matrix}\;\mathrm{effect}=\frac{A_{\mathrm{spiked}\;\mathrm{sample}\left(3\right)}-A_{\;\mathrm{sample}\left(1\right)}}{A_{\mathrm{standard}\;\mathrm{solution}\left(2\right)}}\times 100\%$$

As can be seen from Table S1, all matrix effect values varied from 88.9% to 97.2% with a relative standard deviation (RSD) of less than 5%. The results indicated that there was no significant matrix interference from the concomitant compounds in these samples.

All calibration curves were performed in accordance with linear regression equation of peak areas (*Y*-axis) versus concentrations (*X*-axis, µg/mL) for injection of six standard solutions of appropriate concentrations. All the polyphenols showed good linearity in the range of 0.08–5.33 µg/mL. The correlation coefficients (R^2^) were greater than 0.990 for all analytes. LOD refers to the minimum concentration that could be detected in a sample, whereas, LOQ was the minimum quantity that could be quantitatively determined in a sample. The LOD was considered to be the lowest concentration with a signal-to-noise ratio (S/N) = 3. The LOQ was set as the lowest concentration with S/N = 10. The range for LOD and LOQ were 0.61–103.13 ng/mL and 2.05–343.77 ng/mL, respectively. The regression equations, calibration range, R^2^, LOD and LOQ for the analysis of the 11 compounds are shown in Table 2. The data shown in Table 2 indicate that for the 11 compounds, the LOD of quercitrin was the lowest and that of vanillin was the highest. As a result, the sensitivity for quercitrin was better than for the others when using ESI^−^.

Precision refers to the degree of similarity of multiple sampling results within the same uniform sample. Precision is usually expressed in terms of relative standard deviation (RSD). In order to evaluate the precision of the proposed method, six replicate samples at the same concentration levels were analyzed within one day for intra-day precision, and within three consecutive days for inter-day precision. RSDs of the measured results (intra- and inter-day) were less than 6.9% and 7.9%, respectively. As shown in Table 3, low RSD ensured good precision.

In order to evaluate the accuracy of the UPLC-ESI-MS/MS method, the extraction recovery was characterized using *Moringa oleifera* leaf (S4). Within the specified range, the accuracy was expressed with the results of at least six repetitions of the same concentration (equivalent to 100% concentration level), the content ratio of the standard substance and the component determined in the sample were controlled at 1:1. Standard mixtures (gallic acid, chlorogenic acid, vanillin, ferulic acid, gallogen, rutin, isoquercetin, quercitrin, baicalin, quercetin, and kaempferide) were added to the sample of *Moringa oleifera* extract. Spiked samples were prepared in tuplicate. The percentage recovery of analytes was calculated as follows: recovery (%) = 100 × (amount found − original amount)/amount added. The average recovery of the researched compounds was between 89.2% and 98.2%, and the RSD values were all less than 5.0% (Table 3). The recovery tests indicated that the proposed UPLC-ESI-MS/MS method had good accuracy for the determination of these compounds. The results also demonstrate that the developed method could be used for quality control of *Moringa oleifera*.

Ruggedness means that the test results are not affected by small changes in the test conditions. Ruggedness was evaluated according to the RSD values of six repetitions for each variable factor. and was assessed for variable chromatographic factors at each standard concentration level of 100 ng/mL. The chromatographic parameters were as follows: mobile phase composition ratio (60:40 (*v*/*v*) or 62:38 (*v*/*v*), for acetonitrile, 0.1% formic acid aqueous solution), different brands of columns (ACQUITY UPLC BEH C18 and Agilent Eclipse Plus), column temperatures (30 or 40 °C) and flow rate (0.2 or 0.3 mL/min). The RSD values of the peak areas of compounds for all variable chromatographic factors are shown in Table 3. These values were all less than 5.0%, which demonstrates that the measured results were not influenced by small changes in factors representing the typical variation.

### 2.4. Distribution of Polyphenols in Different Parts of Moringa oleifera

**Figure 2:**
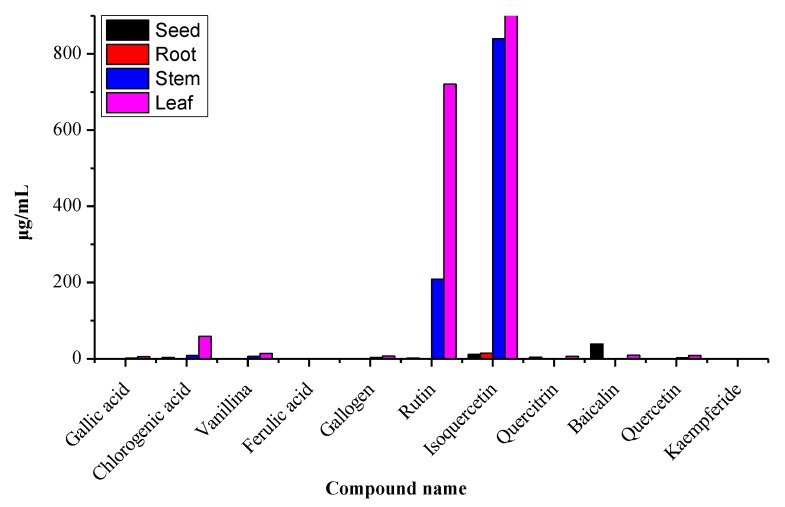
Analysis variation in polyphenol concentrations in the different parts of *Moringa oleifera*.

The presented method was applied to determine the contents of polyphenols in the different plant parts. As can be seen in Figure 2, on one hand, the total contents of the 11 polyphenols for leaves were higher than other parts and, those of stems were second. The abundance of polyphenol contents can be ranked as follows: leaves > stems > seeds > roots. The main reason for this trend is that polyphenols are produced in leaves during photosynthesis, and the stems are important for transport. On the other hand, the isoquercetin content was the highest, and that of rutin was the second highest. The number of components varied greatly due to differences in ecological factors and environmental conditions.

### 2.5. Sample Analysis

Given that the polyphenol content in *Moringa oleifera* leaves were higher than that of the other parts, the presented method was applied to determine the contents of *Moringa oleifera* leaves (S1–S25) collected from different locations in Yunnan province. As shown in Table 4 and Table 5, the contents of isoquercetin, rutin and chlorogenic acid were much higher than those of other polyphenols. These results are consistent with the results described in previous studies [34,38,39]. At the same time, kaempferide and baicalin levels were below the detection limit. This may account for the noticeable lack of interest in these two analytes in previous studies. Of all 25 samples, gallic acid (6.26 μg/g) and ferulic acid (1.59 μg/g) were the highest in sample S10; the values were possibly affected by a variety of factors. Chlorogenic acid (152.52 μg/g) in sample S24, which was the highest value, was influenced by low altitude. Due to high altitude and lower annual mean temperature, vanillin (26.19 μg/g), gallogen (17.29 μg/g), and quercetin (18.34 μg/g) were higher in sample S25 than in the others. Rutin (858.75 μg/g) and quercitrin (3.08 μg/g) in sample S21 were higher than for the other samples due to higher altitude and the highest annual precipitation. Isoquercetin in sample S1, which had the highest value at 1575.28 μg/g, was mainly influenced by lower annual precipitation. Baicalin (1.86 μg/g) in sample S19 was the highest; this value was likely affected by many factors. Kaempferide was very limited in *Moringa oleifera* leaves in samples S1–S25. The greatly variation observed in the levels of these compounds is likely due to differences in sample sources, environmental conditions, and collection times. As a result, the proposed method was simple, fast, accurate, and precise technique for qualitative and quantitative measurements of polyphenols in *Moringa oleifera* leaves.

### 2.6. Hierarchical Cluster Analysis (HCA)

**Figure 3:**
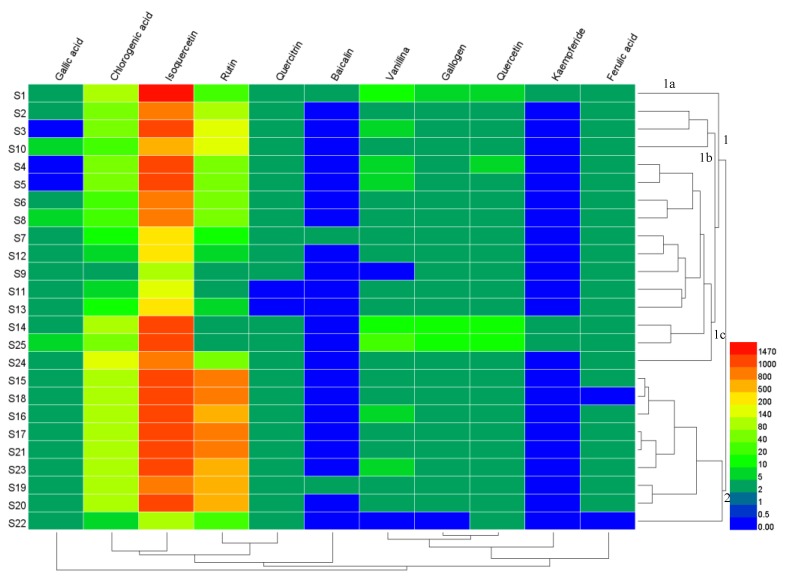
The heatmap of content differences of samples, as well as a dendrogram showing the hierarchical cluster analysis (HCA) results for the concentrations of the 11 components in 25 batches of samples.

HCA was used for the qualitative assessment of the contents of 11 compounds in *Moringa oleifera* leaves to investigate the similarities and differences for samples S1–S25. Average linkage hierarchical clustering was applied to build up the cluster. An 11 × 25 matrix was established using data for the contents of the 11 compounds from samples S1–S25, and the magnitude of all values is represented using thermography. Figure 3 shows the results of HCA, and the content variance of each component can be clearly seen. The horizontal axis represents the compounds and the vertical axis represents the samples. The color shade reveal the content level; red indicates that the compound content in the samples was higher than the average value; blue shows that the compound content was lower than the average value. The content of isoquercetin in all samples was the highest, while that of kaempferide was the lowest.

According to the measurable features of the 11 researched compounds, it was clear that the samples could be divided into two clusters, namely samples S15–S23 in cluster 2 which primarily came from Dehong, and samples S1–S14, S24, and S25 in cluster 1. Cluster 1 could be further classified into subcluster 1a, 1b and 1c. As seen in Figure 3, sample S1 in subcluster 1a was from Baoshan, and samples S2, S3, and S10 could be grouped into subcluster 1b from Baoshan and Dehong. Subcluster 1c comprised samples S4–S9, S11–S14 and S24–S25, and was mainly from Dehong. Additionally, samples from Dehong were classified into cluster 2, and subcluster 1b and 1c. Samples from Kunming and Xishuangbanna were categorized into subcluster 1c. Samples from Nujiang were divided into cluster 2. The classifications were based purely on the change in contents of the measured components.

### 2.7. Principal Component Analysis (PCA)

**Figure 4:**
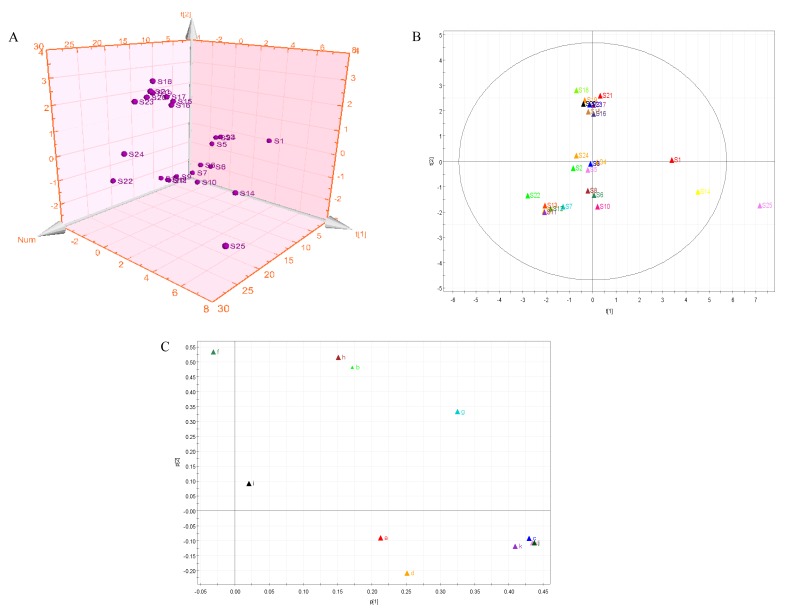
(**A**) The 3D scatter plot of scores of samples from the principle component analysis (PCA) model. (**B**) PCA score plot of 25 *Moringa oleifera* samples. (**C**) The loading scatter plot of the PCA model (a: gallic acid; b: chlorogenic acid; c: vanillin; d: ferulic acid; e: gallogen; f: rutin; g: isoquercetin; h: quercitrin; i: baicalin; j: quercetin; and k: kaempferide).

PCA was used to reveal the relationship between multiple variables through research of a few major components. Twenty-five samples from different areas were taken as observations, and the amounts of the 11 chemical components were taken as variables. The resulting score scatter plot is shown as a three-dimensional PCA model (Figure 4A). Figure 4A shows that the score distribution of samples is obviously scattered according to the different areas. Nevertheless, the scores of samples S15–S21, and S23 clustered together, which illustrates that the chemical components and content matter of the respective samples were very similar. On the other hand, the scatter scores of samples that were far apart indicated that the similarity was small. The results of this analysis are in accordance with those of HCA.

The near-far distance represented the difference of chemical compositions in different areas; as shown in Figure 4B, samples S15–S21 and S23 of the PCA score plot were the closest, which indicated the closest chemical composition. Samples S1, S14, and S25 were far away from other samples, which indicates a large difference in chemical components. Figure 4C shows that compounds b (chlorogenic acid), c (vanillin), e (gallogen), f (rutin), g (isoquercetin), h (quercitrin), j (quercetin), and k (kaempferide) had the maximum loading coefficients, which indicated that these eight compounds had more significant effects on sample origin differentiation.

As expected, *Moringa oleifera* leaves collected from different locations in Yunnan province presented differences in quantitative polyphenol content as a consequence of different environmental conditions, genetic factors, cultivation methods, and drying techniques. Forster et al. [40] reported high ecotype variability in the secondary metabolite content in *Moringa oleifera* leaves. In addition, the authors observed that environmental factors such as water deficiency and sunlight intensity determined an increment of phenolic compound production. However, we cannot exclude the existence of different ecotypes of *Moringa oleifera* in these locations. Certainly, these findings, which are in agreement with those of other authors [41,42], reinforce the importance of characterizing the phenolic characteristics of *Moringa oleifera* leaves before using them for nutritional and pharmacological purposes.

## 3. Materials and Methods

### 3.1. Chemicals and Reagents

Gallic acid, chlorogenic acid, vanillin, ferulic acid, gallogen, rutin, isoquercetin, quercitrin, baicalin, quercetin, and kaempferide were purchased from the National Institute for the Control of Pharmaceutical and Biological Products (Beijing, China). Deionized water was obtained from a Milli-Q filtration system (Millipore Filter Co., Bedford, MA, USA). The LC/MS grade acetonitrile and formic acid were purchased from Fisher (Thermo Fisher Scientific Inc., Waltham, MA, USA). Other chemicals and solvents (analytical grade) were purchased from Zhiyuan Chemical Factory (Tianjin, China).

### 3.2. Preparation of Standard Solutions

Standard stock solutions of gallic acid, chlorogenic acid, vanillin, ferulic acid, gallogen, rutin, isoquercetin, quercitrin, baicalin, quercetin, and kaempferide were prepared in methanol at 0.3 mg/mL. Working standard solutions were made by diluting individual standard stock solutions with methanol. The solutions were kept away from light because the 11 compounds containing phenolic hydroxyl groups oxidize easily. The stock and working standard solutions were stored at −20 °C before use. All solutions were filtered through a 0.22 μm membrane filter before UPLC-ESI-MS/MS analysis.

### 3.3. Liquid Chromatographic Conditions

This research was performed on an Acquity I-class UPLC system (Waters Corp., Milford, MA, USA) equipped with a controller, two pumps, a degasser, and an autosampler. Separation of polyphenols was accomplished on an ACQUITY UPLC BEH C18 column (100 mm × 2.1 mm, 1.7 μm) maintained at 30 °C. The mobile phase consisted of acetonitrile (A) and 0.1% formic acid aqueous solution (B) using a linear gradient program of 5%–100% (A) for 0.0–4.0 min, 100%–90% (A) for 4.0–4.1 min, 90% (A) for 4.1–5.0 min, 90%–5% (A) for 5.0–5.1 min and 5% (A) for 5.1–6.0 min. The flow rate was 0.3 mL/min, and the injection volume was 1 μL. The autosampler was set at 10 °C.

### 3.4. MS/MS Conditions

A Xevo tandem quadruple detector (TQD) mass spectrometer was operated in negative ionization mode (ESI^−^) (Waters Corp., Milford, MA, USA). Quantitative analysis was achieved in the multiple reaction monitoring (MRM) mode. The MRM conditions were optimized for each compound by direct infusion of reference standards using a syringe pump prior to sample analysis. In order to acquire maximum signals for polyphenols, the optimized TQD tuning parameters were as follows: capillary voltage, 2.0 kV; cone voltage, 36 V; source temperature, 120 °C; desolvating temperature, 500 °C; source desolvating gas flow, 1000 L/h; and cone gas flow, 50 L/h. The optimal MRM conditions (precursor/product, cone voltage, collision energy, and retention time) were determined by injecting the analytes into the mass spectrometer; each compound had two different products. The MRM conditions are shown in Table 1**.**

### 3.5. Sample Preparation

**Figure 5:**
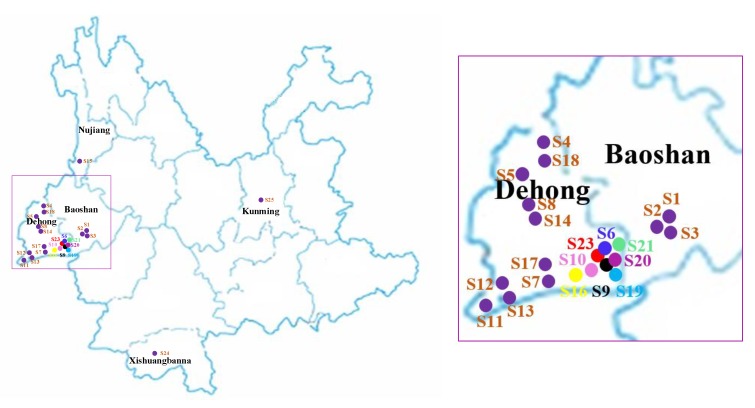
The sample collection areas of *Moringa oleifera* on the map.

The samples collected from February to March 2018 from Yunnan province are listed in Table 4, and a map of their places of production is provided in Figure 5. Each sample was crushed, passed through a 100 mesh sieve, and stored in a natural-draft drier. One gram of each sample powder was accurately weighed and adequately extracted by an ultrasonic cleaner at 20 °C for 40 min with 100% methanol. The extracted solutions were filtered through a 0.22 μm filter membrane before injection. Finally, 1 μL of the solution was injected in the UPLC-ESI-MS/MS system for analysis.

### 3.6. Data Processing and Analysis

Data were obtained using MassLynx software (version 4.1) and processed using the TargetLynx program. Variation in the contents of 25 sample batches of *Moringa oleifera* from different regions were analyzed. PCA and HCA were conducted using Simca-P + 11.5 (Umetrics Company, Umea, Sweden) and MultiExperiment Viewer (version 4.8) software, respectively.

## 4. Conclusions

In conclusion, the UPLC-ESI-MS/MS method was established and validated for the simultaneous quantitative determination of polyphenols in the extracts of *Moringa oleifera*. The UPLC-ESI-MS/MS method was simple, rapid, efficient and reliable. Moreover, the changes of 11 phenolic components were further studied by hierarchical cluster analysis (HCA) and principal component analysis (PCA). Consequently, a significant correlation was found between the composition content and the collection area. The quantitative analysis methodology of polyphenols and their applications to the pharmacokinetic profile can promote the quality assessment and future pharmacodynamics investigation of *Moringa oleifera*, providing important potential therapeutic knowledge for functional food.

## Figures and Tables

**Table 1:** The multiple reaction monitoring (MRM) conditions of 11 polyphenols.

Compound Name	Precursor (*m*/*z*)	Product (*m*/*z*)	Cone (V)	Collision (eV)	Retention Time (min)	Remark
Gallic acid	168.96	125.04	36	14	1.37	Quantifier
	168.96	79.07	36	22	1.37	Qualifier
Chlorogenic acid	353.09	191.11	34	20	1.72	Quantifier
	353.09	85.12	34	46	1.72	Qualifier
Vanillin	150.99	106.99	60	8	2.61	Quantifier
	150.99	83.06	60	14	2.61	Qualifier
Ferulic acid	193.03	134.09	36	16	2.24	Quantifier
	193.03	178.12	36	14	2.24	Qualifier
Gallogen	301.06	151.11	42	24	2.61	Quantifier
	301.06	179.07	42	16	2.61	Qualifier
Rutin	609.10	300.24	66	36	1.94	Quantifier
	609.10	271.11	66	62	1.94	Qualifier
Isoquercetin	463.03	300.24	44	26	2.03	Quantifier
	463.03	271.12	44	46	2.03	Qualifier
Quercitrin	447.03	300.42	46	24	2.16	Quantifier
	447.03	271.11	46	44	2.16	Qualifier
Baicalin	445.08	269.10	28	20	2.35	Quantifier
	445.08	275.00	28	42	2.35	Qualifier
Quercetin	301.00	151.05	40	24	2.61	Quantifier
	301.00	179.08	40	16	2.61	Qualifier
Kaempferide	298.97	284.13	56	22	3.46	Quantifier
	298.97	151.04	56	30	3.46	Qualifier

**Table 2:** Analytical performance data of polyphenols by UPLC-ESI-MS/MS.

Compound Name	Regression Equation	Calibration Range (µg/mL)	R^2^	LOD (ng/mL)	LOQ (ng/mL)
Gallic acid	Y = 246.765X − 5.02768	0.12–4.81	0.9952	15.29	50.98
Chlorogenic acid	Y = 269.863X + 6.49917	0.09–3.64	0.9921	15.21	50.69
Vanillin	Y = 97.6209X − 2.37704	0.13–5.14	0.9954	103.13	343.77
Ferulic acid	Y = 233.118X − 10.8833	0.13–5.06	0.9952	0.87	2.90
Gallogen	Y = 492.921X − 10.2403	0.10–4.00	0.9968	0.84	2.81
Rutin	Y = 296.566X − 8.78358	0.13–5.33	0.9984	0.65	2.18
Isoquercetin	Y = 210.1X − 3.09922	0.11–4.31	0.9988	0.86	2.86
Quercitrin	Y = 286.726X − 9.29514	0.10–3.77	0.9926	0.61	2.05
Baicalin	Y = 73.2853X − 3.93154	0.12–4.74	0.9916	3.00	9.99
Quercetin	Y = 420.472X − 5.82231	0.11–4.20	0.9942	5.21	17.36
Kaempferide	Y = 2221.11X + 11.1101	0.08–3.34	0.9995	6.78	22.59

**Table 3:** The precision, recovery and ruggedness data for 11 reference compounds.

Compound Name	Precision (*n* = 6) RSD (%)		Ruggedness (RSD%)	Recovery (*n* = 6)	
	Intra-Day	Inter-Day		Mean (%)	RSD (%)
Gallic acid	5.5	3.8	4.3	95.5	1.0
Chlorogenic acid	5.5	6.4	3.8	95.5	3.0
Vanillin	5.3	7.9	4.6	96.8	3.5
Ferulic acid	5.7	6.2	3.4	89.2	3.7
Gallogen	6.9	7.2	2.7	95.7	3.0
Rutin	6.5	6.6	3.2	94.9	2.4
Isoquercetin	6.2	6.4	1.3	93.7	1.4
Quercitrin	6.6	5.0	4.3	98.2	3.2
Baicalin	5.4	4.5	3.0	95.4	2.2
Quercetin	6.1	6.7	2.3	92.6	3.4
Kaempferide	1.4	1.8	1.9	94.5	1.1

**Table 4:** Representative samples of *Moringa oleifera* investigated in this study.

No.	Collecting Area	Sample Source	Altitude (m)	Annual Mean Temperature (°C)	Annual Precipitation (mm)	Part	Collection Time (yy/mm/dd)
S1	Baoshan	Lujiang farm, Baoshan city	758	21	850	Leaf	2018/2/1
S2	Baoshan	Xincheng farm, Baoshan city	758	21	850	Leaf	2018/2/2
S3	Baoshan	Baihua village, Longyang district	758	21	850	Leaf	2018/2/2
S4	Dehong	Zhina township, Yingjiang county	820	19	1464	Leaf	2018/2/3
S5	Dehong	Mengnong township, Yingjiang county	820	19	1464	Leaf	2018/2/4
S6	Dehong	Mangshi Lamu division	807	20	1655	Leaf	2018/2/5
S7	Dehong	Mangshi Zhefang town	807	20	1655	Leaf	2018/2/2
S8	Dehong	Mangshi Laman village	807	20	1655	Leaf	2018/2/3
S9	Dehong	Mangshi Menghuan load	908	20	1655	Leaf	2018/2/5
S10	Dehong	Mangshi Fapa town	884	20	1655	Leaf	2018/2/4
S11	Dehong	Nongdao town, Ruili city	748	21	1395	Leaf	2018/2/5
S12	Dehong	Mengxiu township, Ruili city	778	21	1395	Leaf	2018/2/5
S13	Dehong	Ruili farm, Ruili city	778	21	1395	Leaf	2018/2/5
S14	Dehong	Huguo township, Longchuan county	965	19	1595	Leaf	2018/2/5
S15	Nujiang	Liuku town, Lushui county	869	21	747	Leaf	2018/2/6
S16	Dehong	Padi industrial district, Mangshi	874	20	1655	Leaf	2018/2/6
S17	Dehong	Xishan township, Mangshi	933	20	1655	Leaf	2018/2/6
S18	Dehong	Mangzhang township, Yingjiang county	820	19	1464	Leaf	2018/2/6
S19	Dehong	Mangjiu reservoir, Mangshi	888	20	1655	Leaf	2018/3/2
S20	Dehong	Mangshi town, Mangshi	911	20	1655	Leaf	2018/3/2
S21	Dehong	South-sky gate, Mangshi	1203	20	1655	Leaf	2018/3/2
S22	Dehong	Menghuan load B, Mangshi	908	20	1655	Leaf	2018/3/2
S23	Dehong	Menghuan load A, Mangshi	908	20	1655	Leaf	2018/3/3
S24	Xishuangbanna	Menghan town, Jinghong city	527	22	1068	Leaf	2018/3/3
S25	Kunming	Chenggong district, Kunming city	1903	15	1035	Leaf	2018/3/3

**Table 5:** The contents of *Moringa oleifera* leaves collected from different locations in Yunnan province (*n* = 3).

No.	Content (μg/g)										
	Gallic Acid	Chlorogenic Acid	Vanillin	Ferulic Acid	Gallogen	Rutin	Isoquercetin	Quercitrin	Baicalin	Quercetin	Kaempferide
S1	2.00 ± 0.02	110 ± 2	11.7 ± 0.4	1.20 ± 0.06	5.58 ± 0.28	32.64 ± 3.59	1575 ± 40	1.61 ± 0.08	0.82 ± 0.02	6.69 ± 0.33	0.01 ± 0.001
S2	0.20 ± 0.02	45.5 ± 0.7	1.72 ± 0.05	0.53 ± 0.01	3.00 ± 0.15	133 ± 14	996 ± 35	0.93 ± 0.05	ND	2.62 ± 0.13	ND
S3	ND	65.9 ± 1.0	5.92 ± 0.20	0.69 ± 0.02	3.91 ± 0.20	187 ± 21	1134 ± 28	0.86 ± 0.04	ND	3.21 ± 0.16	ND
S4	ND	69.3 ± 0.2	7.46 ± 0.09	0.72 ± 0.02	3.21 ± 0.16	58.2 ± 6.4	1172 ± 42	1.34 ± 0.07	ND	4.12 ± 0.21	ND
S5	ND	63.6 ± 1.3	5.87 ± 0.12	0.54 ± 0.01	3.59 ± 0.18	42.6 ± 4.7	1057 ± 16	0.84 ± 0.04	ND	3.90 ± 0.20	ND
S6	3.88 ± 0.09	36.3 ± 0.2	3.36 ± 0.05	1.41 ± 0.06	2.35 ± 0.12	73.0 ± 8.0	975 ± 24	0.54 ± 0.03	ND	1.43 ± 0.07	ND
S7	1.59 ± 0.06	10.8 ± 0.3	2.39 ± 0.08	1.26 ± 0.06	0.54 ± 0.03	13.4 ± 1.5	341 ± 9	0.34 ± 0.02	0.91 ± 0.03	0.56 ± 0.03	ND
S8	4.24 ± 0.10	30.4 ± 0.7	3.07 ± 0.07	1.08 ± 0.05	1.61 ± 0.08	53.8 ± 5.9	860 ± 22	0.89 ± 0.04	ND	1.96 ± 0.10	ND
S9	0.74 ± 0.04	2.91 ± 0.09	ND	0.96 ± 0.05	0.26 ± 0.01	2.52 ± 0.28	115 ± 3	0.40 ± 0.02	ND	0.14 ± 0.01	ND
S10	6.26 ± 0.13	25.4 ± 0.8	3.63 ± 0.06	1.59 ± 0.07	1.55 ± 0.08	143 ± 16	670 ± 17	0.58 ± 0.03	ND	1.93 ± 0.10	ND
S11	0.55 ± 0.01	4.66 ± 0.05	1.71 ± 0.02	0.75 ± 0.02	0.36 ± 0.02	1.51 ± 0.17	191 ± 5	ND	ND	0.23 ± 0.01	ND
S12	1.12 ± 0.07	5.41 ± 0.13	0.93 ± 0.04	0.91 ± 0.05	0.44 ± 0.02	7.53 ± 0.83	241 ± 6	0.38 ± 0.02	ND	0.66 ± 0.03	ND
S13	0.40 ± 0.02	11.0 ± 0.2	1.57 ± 0.05	0.45 ± 0.01	1.03 ± 0.05	5.34 ± 0.59	263 ± 7	ND	ND	1.07 ± 0.05	ND
S14	2.10 ± 0.09	83.3 ± 2.7	10.0 ± 0.4	1.34 ± 0.06	13.0 ± 0.65	1.94 ± 0.21	1302 ± 33	1.36 ± 0.07	ND	13.5 ± 0.7	0.01 ± 0.002
S15	1.14 ± 0.03	121 ± 3	2.29 ± 0.11	0.93 ± 0.05	1.21 ± 0.06	839 ± 92	1180 ± 29	1.87 ± 0.09	ND	1.80 ± 0.09	ND
S16	1.51 ± 0.04	103 ± 4	4.21 ± 0.15	0.98 ± 0.05	1.77 ± 0.09	750 ± 83	1037 ± 26	2.69 ± 0.13	ND	1.55 ± 0.08	ND
S17	1.83 ± 0.08	118 ± 1	3.43 ± 0.09	0.79 ± 0.04	1.75 ± 0.09	817 ± 90	1178 ± 42	2.51 ± 0.13	ND	1.55 ± 0.08	ND
S18	1.37 ± 0.06	115 ± 1	0.72 ± 0.01	ND	1.38 ± 0.07	826 ± 91	1154 ± 27	2.76 ± 0.14	ND	2.16 ± 0.11	ND
S19	1.85 ± 0.05	114 ± 3	1.76 ± 0.04	0.57 ± 0.01	1.79 ± 0.09	753 ± 83	992 ± 25	2.36 ± 0.12	1.86 ± 0.05	1.35 ± 0.07	ND
S20	1.49 ± 0.02	117 ± 4	2.52 ± 0.17	0.57 ± 0.01	1.32 ± 0.07	791 ± 87	1056 ± 26	2.53 ± 0.13	ND	1.15 ± 0.06	ND
S21	1.76 ± 0.10	122 ± 3	3.24 ± 0.22	0.96 ± 0.05	1.84 ± 0.09	859 ± 54	1242 ± 31	3.08 ± 0.15	ND	2.05 ± 0.10	ND
S22	0.20 ± 0.07	6.56 ± 0.27	ND	ND	ND	36.0 ± 4.0	103 ± 6	0.37 ± 0.02	ND	0.14 ± 0.02	ND
S23	1.61 ± 0.06	115 ± 2	4.38 ± 0.30	0.67 ± 0.02	1.51 ± 0.08	764 ± 84	1087 ± 27	2.73 ± 0.14	ND	0.98 ± 0.05	ND
S24	0.56 ± 0.04	153 ± 5	0.72 ± 0.08	1.04 ± 0.05	0.70 ± 0.04	51.2 ± 5.6	846 ± 21	0.63 ± 0.03	ND	1.28 ± 0.06	ND
S25	4.24 ± 0.16	77.2 ± 2.4	26.2 ± 2.5	1.43 ± 0.06	17.3 ± 0.9	2.95 ± 0.32	1319 ± 33	1.73 ± 0.09	ND	18.3 ± 0.9	0.01 ± 0.001

Values are mean ± SD (*n* = 3). ND, not detected.

## Supplementary Materials

The following are available online. Figure S1: Precursors of 11 polyphenols in TQD MS (a: gallic acid; b: chlorogenic acid; c: vanillin; d: ferulic acid; e: gallogen; f: rutin; g: isoquercetin; h: quercitrin; i: baicalin; j: quercetin; and k: kaempferide). Figure S2: The effect of extraction solvents, extraction method, extraction time, and temperature in the extraction procedure (a: extraction solvents; b: extraction method; c: extraction time; and d: temperature).Table S1: Matrix effects and their precision (RSD) of the proposed method in *Moringa oleifera* leaves spiked at 0.5 μg/mL level.
