# Supplementary material for: Comprehensive Investigation of Moringa oleifera from Different Regions by Simultaneous Determination of 11 Polyphenols Using UPLC-ESI-MS/MS
Source: Molecules. 2020 Feb 5;25(3):676. doi: 10.3390/molecules25030676 (PMC7037984; doi:10.3390/molecules25030676)
Supplement: Supplementary file 1 [file molecules-25-00676-s001.pdf]

SUPPLEMENTARY MATERIALS

Comprehensive Investigation of *Moringa oleifera* from Different Regions by Simultaneous Determination of 11 Polyphenols Using UPLC-ESI-MS/MS

Yanqin Zhu [1,2], Qinhong Yin [3] and Yaling Yang [1,*]

[1] Faculty of Life Science and Technology, Kunming University of Science and Technology, Kunming 650500, Yunnan, China
[2] Research Center for Analysis and Measurement, Kunming University of Science and Technology, Kunming 650093, China
[3] Yunnan Police College, Kunming 650223, China
* Correspondence: ygs2013@yeah.net; Tel.: +86-871-65113971

SUPPLEMENTARY MATERIALS CONTENT

Figure S1. Precursors of 11 polyphenols in TQD MS (a: gallic acid, b: chlorogenic acid, c: vanillina, d: ferulic acid, e: gallogen, f: rutin, g: isoquercetin, h: quercitrin, i: baicalin, j: quercetin and k: kaempferide).

Figure S2. The effect of extraction solvents, extraction method, extraction time and temperature in the extraction procedure (a: extraction solvents, b: extraction method, c: extraction time, d: temperature).

Table S1. Matrix effects and their precision (RSD) of the proposed method in *Moringa oleifera* leaves matrices spiked at 0.5 μg/mL level.

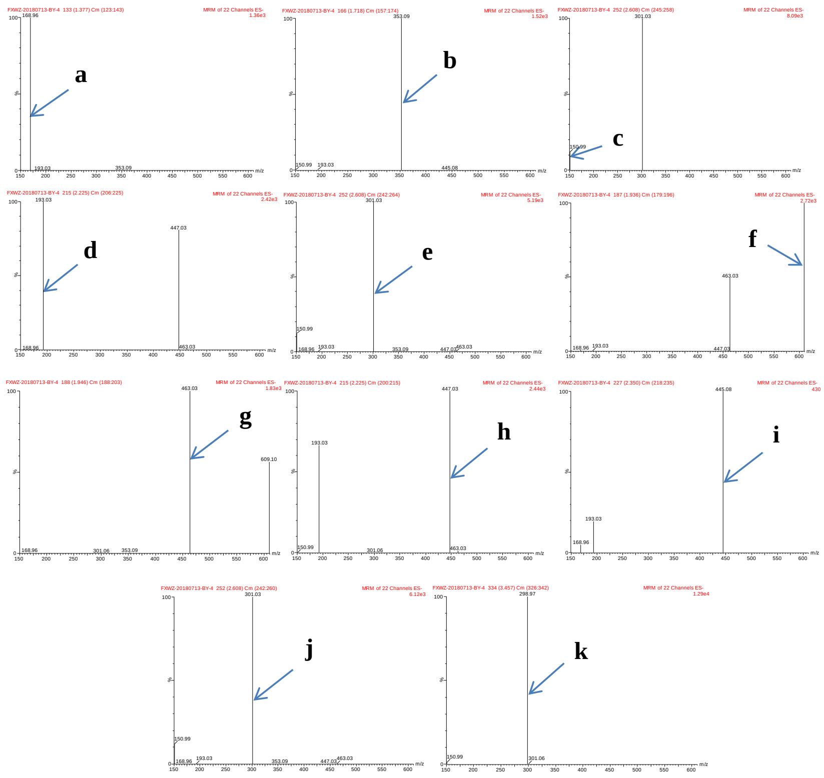

Figure S1. Precursors of 11 polyphenols in TQD MS (a: gallic acid, b: chlorogenic acid, c: vanillina, d: ferulic acid, e: gallogen, f: rutin, g: isoquercetin, h: quercitrin, i: baicalin, j: quercetin and k: kaempferide).

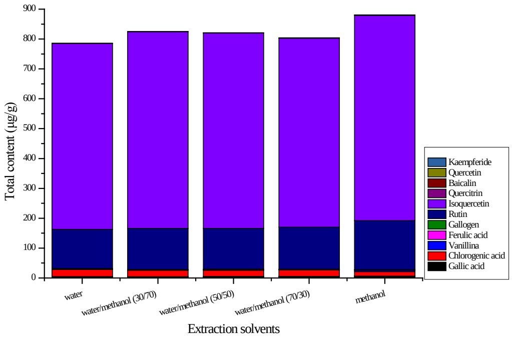

(a)

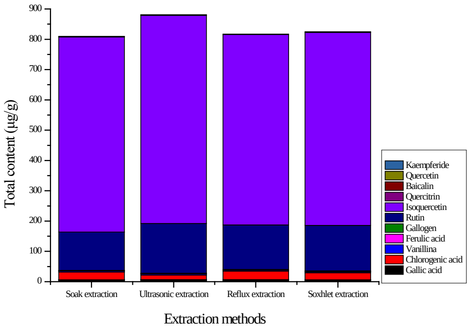

(b)

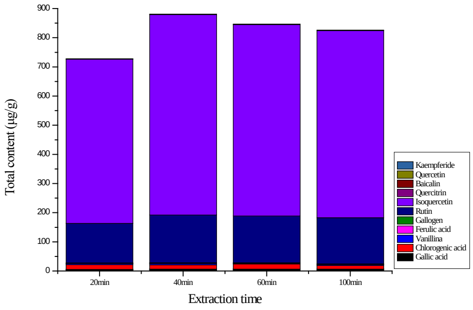

(c)

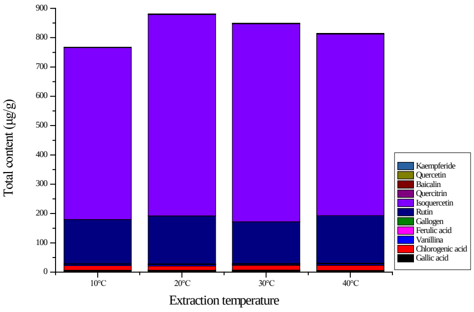

(d)

Figure S2. The effect of extraction solvents, extraction method, extraction time and temperature in the extraction procedure (a: extraction solvents, b: extraction method, c: extraction time, d: temperature).

Table S1

Matrix effects and their precision (RSD) of the proposed method in *Moringa oleifera* leaves matrices spiked at 0.5 μg/mL level.

Compound Name	Moringa Leaves	
	Matrix Effect (%)	RSD (%)
Gallic acid	92.3	4.1
Chlorogenic acid	96.0	3.5
Vanillin	96.5	4.8
Ferulic acid	92.3	3.2
Gallogen	93.8	3.5
Rutin	88.9	3.0
Isoquercetin	97.2	4.0
Quercitrin	93.2	1.7
Baicalin	97.8	2.3
Quercetin	94.4	2.8
Kaempferide	90.7	3.9
